# Seroprevalence and Risk Factors for Exposure to Equine Coronavirus in Apparently Healthy Horses in Israel

**DOI:** 10.3390/ani11030894

**Published:** 2021-03-21

**Authors:** Gili Schvartz, Sharon Tirosh-Levy, Samantha Barnum, Dan David, Asaf Sol, Nicola Pusterla, Amir Steinman

**Affiliations:** 1 Koret School of Veterinary Medicine, The Robert H. Smith Faculty of Agriculture, Food and Environment, The Hebrew University of Jerusalem, Rehovot 7610001, Israel; giliun@gmail.com (G.S.); sharontirosh@gmail.com (S.T.-L.); 2 Division of Virology, Kimron Veterinary Institute, Beit Dagan 50250, Israel; Dand@moag.gov.il (D.D.); Asafs@moag.gov.il (A.S.); 3 Department of Medicine and Epidemiology, School of Veterinary Medicine, University of California, Davis, CA 95616, USA; smmapes@ucdavis.edu (S.B.); npusterla@ucdavis.edu (N.P.)

**Keywords:** equine coronavirus, horse, enteric disease, ECoV, seroprevalence

## Abstract

**Simple Summary:**

Equine coronavirus (ECoV) is a *β-coronavirus* that, together with other coronaviruses, are pathogenic to both human and animals, as seen in the recent COVID-19 pandemic. ECoV is considered as a diarrheic pathogen in foals and is included in the list of viral causes of enteritis. During the last decade, outbreaks of ECoV were reported in adult horses in the USA, EU and Japan. In Israel, other coronaviruses were reported in cattle, camels and in humans; however, coronaviruses have not been reported in horses. In this study, we aimed to determine the exposure of healthy horses to ECoV and determine the selected risk factors for infection. For this purpose, serum samples were collected from 333 healthy horses, 41 (12.3%) of which had anti-ECoV antibodies. Seropositive horses were found in more than half (58.6%) of the farms and horses located in central Israel were more likely to be positive. ECoV should be included in the differential diagnosis list of pathogens in cases of adult horses with acute onset of anorexia, lethargy, fever and gastrointestinal signs in Israel.

**Abstract:**

Equine coronavirus (ECoV) infection is the cause of an emerging enteric disease of adult horses. Outbreaks have been reported in the USA, EU and Japan, as well as sporadic cases in the UK and Saudi Arabia. Infection of ECoV in horses in Israel has never been reported, and the risk of exposure is unknown. Importation and exportation of horses from and into Israel may have increased the exposure of horses in Israel to ECoV. While the disease is mostly self-limiting, with or without supportive treatment, severe complications may occur in some animals, and healthy carriers may pose a risk of infection to other horses. This study was set to evaluate the risk of exposure to ECoV of horses in Israel by using a previously validated, S1-based enzyme-linked immunosorbent assay (ELISA). A total of 41 out of 333 horses (12.3%) were seropositive. Exposure to ECoV was detected in 17 of 29 farms (58.6%) and the seroprevalence varied between 0 and 37.5% amongst farms. The only factor found to be significantly associated with ECoV exposure in the multivariable model was the geographical area (*p* < 0.001). ECoV should be included in the differential diagnosis list of pathogens in cases of adult horses with anorexia, lethargy, fever and gastrointestinal signs in Israel.

## 1. Introduction

Equine coronavirus (ECoV) is a positive single-stranded RNA enveloped virus, belonging to the family *Coronaviridae.* ECoV is a *β-coronavirus*, together with human coronaviruses OC43, 4408 and HKU1, bovine coronavirus (BCoV), porcine hemagglutinating encephalomyelitis virus, canine respiratory coronavirus, and others [1]. Important other members of this group, which are pathogenic to humans, are Severe Acute Respiratory Syndrome Coronavirus (SARS-CoV), Middle-East Respiratory Syndrome Coronavirus (MERS-CoV) and the recent SARS-CoV-2 (the causative agent of COVID-19) [2]. Coronaviruses may cause enteric or respiratory disease in mammalian and avian species [3,4]. ECoV is considered as a diarrheic pathogen in foals and is included in the list of viral causes of enteritis together with rotavirus, adenovirus and parvovirus [5]. In the last decade, ECoV has been associated with outbreaks of enteric disease in adult horses in the United States of America, Europe and Japan [6,7,8,9,10,11].

Like many other viral diseases, ECoV infection is mostly self-limiting, but supportive care is often required. The most frequent clinical signs pooled from 20 outbreaks in the USA were anorexia (97%), lethargy (88%) and fever (83%) [12]. Possible complications, including necrotizing enteritis, endotoxemia and hyperammonemia-associated encephalopathy, have been reported [4,7,13]. Morbidity rates vary between 10% and 80% [3,10] and mortality rates are usually low [3,10,14], but reached 27% in one outbreak in American miniature horses [15]. Healthy adult horses may shed the virus in the environment via feces, and orofecal route is considered to be the main source of infection to other horses [11,13]. ECoV may be detected by electron microscopy, cell culture isolation and polymerase chain reaction (PCR) [4,11,13]. Serology is based on the detection of antibodies against the ECoV S1 protein using enzyme-linked immunosorbent assay (ELISA) [16,17]. Since 2010, the number of positive cases has steadily increased in the USA, which is probably driven by increased awareness and testing [12]. In the USA, the seroprevalence in healthy horses from 18 states was 9.6%, with the geographic region and specific uses of horses identified as significant risk factors for seropositivity [9]. Prevalence of ECoV in adult horses in other countries is generally unknown. In France, among horses with either respiratory or gastrointestinal signs, 11/395 and 1/200 fecal samples and nasal swabs, respectively, were positive for ECoV by qPCR [6]. In Saudi Arabia and Oman, 5/316 and 0/306 rectal and nasal swabs, respectively, were positive for ECoV by qPCR [4].

To the best of our knowledge, ECoV has never been reported in horses in Israel. In recent years, other members of the group *β-coronavirus* have been detected in Israel in both animals and human. Antibodies against MERS-CoV were tested in sera samples that were collected from dromedary camels (*Camelus dromedaries*) between 2012 and 2017 and 61.8% had neutralizing antibodies against MERS-CoV (in virus neutralizing test, VNT) [18]. In samples that were collected from influenza-like illness patients in Israel in 2015–2016, HCoV-OC43, HCoV-NL63 and HCoV-229E were detected; however, no MERS-CoV infections were detected in human patients [19]. According to the Israeli veterinary services annual report in Hebrew, more recently, in 2019, 88/264 (33.3%) serum samples from camels were seropositive to MERS-CoV by ELISA, but 0/18 nasal swabs were positive by qPCR [20]. In 2019, 81/245 (33.1%) samples from cattle were also positive for BCoV by qPCR [20]. During the last year, like most countries in the world, Israel experienced a massive COVID-19 outbreak in which hundreds of thousands were infected and more than 5000 humans died. The aim of this study was to investigate the seroprevalence and selected risk factors for infection with ECoV among apparently healthy horses in Israel.

## 2. Materials and Methods

### 2.1. Study Population

Active surveillance and sera collection were conducted in 2018 and included 333 apparently healthy horses from 29 farms throughout Israel (4–32 at each farm) (Table S1). Horse owners approved the sample collection and the study protocol was approved by the Internal Research Committee of the Koret School of Veterinary Medicine–Veterinary Teaching Hospital (KSVM-VTH/08_2017). Fifteen farms and 150 horses were located in northern Israel, six farms with 93 horses were from central Israel and eight farms with 90 horses were from southern Israel. Almost half of the horses were mixed breeds (156, 46.8%) and others were of various breeds, including Quarter horses (65, 19.5%), Arabians (45, 13.5%), Ponies (19, 5.7%), Warmbloods (16, 4.8%) and Tennessee Walking horses (12, 3.6%). The study population included 161 mares (48.3%), 164 geldings (49.2%) and 8 stallions (2.4%). The horses’ age ranged between six months and 47 years (mean = 11.66, median = 11, interquartile range (IQR) = 7). Most horses were kept in stalls (136, 40.8%), some were turned out in paddocks (133, 39.9%) and others were kept on pastures (19.2%, 64).

### 2.2. Sample Collection and Serologic Detection of ECoV Exposure

Blood samples were collected into a sterile tube without anticoagulant from the jugular vein. Serum was separated from each sample after centrifuging at 3000× *g* for 10 min and stored in −20 °C until analysis. Sera were shipped on ice and tested in the Department of Medicine and Epidemiology, School of Veterinary Medicine, University of California, Davis, CA, USA for the presence of antibodies against ECoV using the S1-based enzyme-linked immunosorbent assay (ELISA), as was previously described [16]. The S1-based ELISA targets antibodies to the spike (S) protein of ECoV and has been developed and validated using serum samples from naturally infected adult horses involved in contemporary outbreaks [16].

### 2.3. Statistical Analysis

Risk factors associated with exposure to ECoV (geographical area, horses’ breed and sex and housing management) were assessed by using a chi-square test or two-sided Fisher’s exact test, as appropriate, and the odds ratios were calculated. Association between animal age and exposure to ECoV was evaluated using a non-parametric Mann–Whitney U test. Factors that were found to be significantly associated with ECoV exposure in the univariable analysis were included in a multivariable analysis using the generalized estimating equation (GEE) with the farm set as a subject (i.e., random variable). The analysis was performed using SPSS 25.0^®^ (IBM Corp, Armonk, NY, USA) and Win Pepi 11.65^®^ statistical software (Abramson, J.H. WINPEPI updated: computer programs for epidemiologists, and their teaching potential. Epidemiologic Perspectives & Innovations, 2011, 8:1). Statistical significance was set as *p* < 0.05. A seroprevalence map was prepared using ArcGIS Desktop 10.6.19270 (ESRI, Redlands, CA, USA).

## 3. Results

### 3.1. Equine Coronavirus (ECoV) Seroprevalence

**Figure 1:**
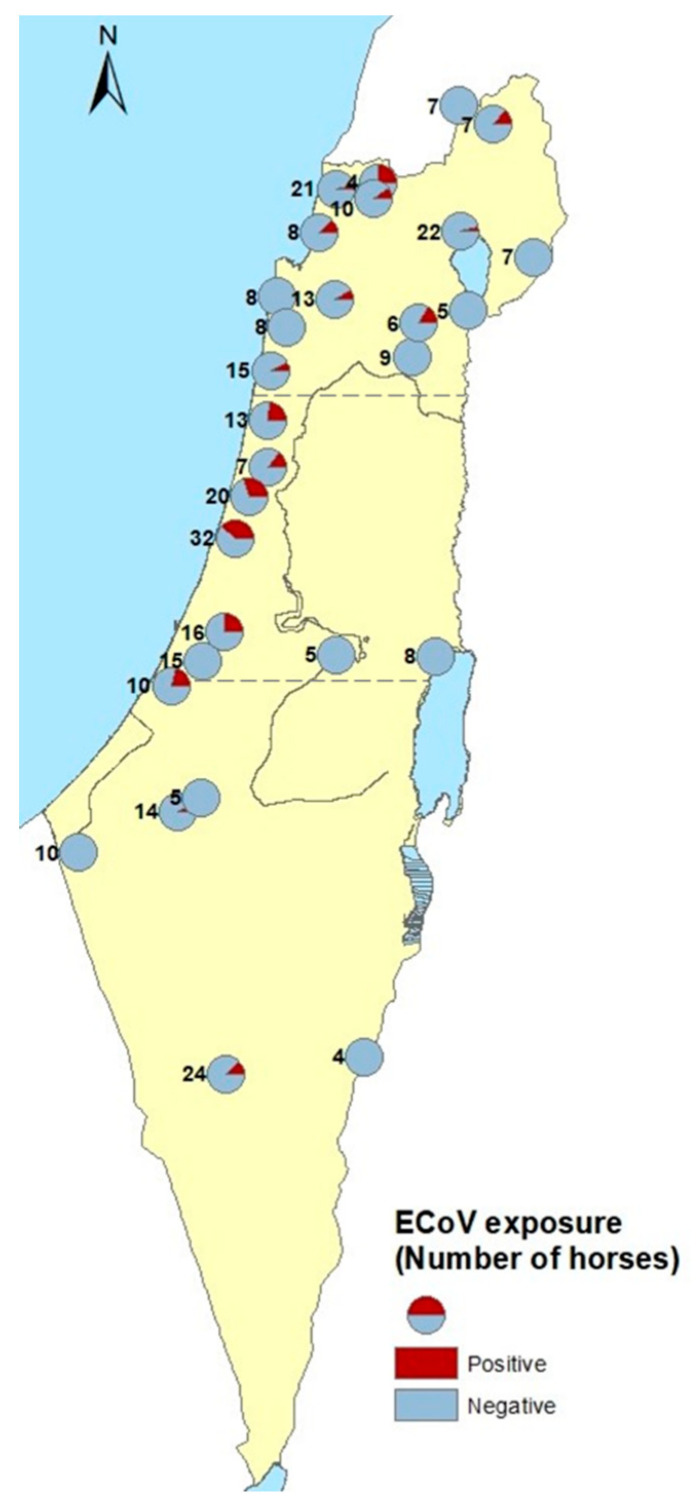
Geographical distribution of farms with horses tested for antibodies against Equine coronavirus (ECoV), indicating farms with only negative or ≥1 positive horse.

The seroprevalence of ECoV in the study population was 12.3% (95% confidence interval (CI): 8.98–16.33%), with 41 of the 333 horses testing positive for the presence of anti-ECoV antibodies. The seroprevalence in different farms varied between 0 and 37.5%. Exposure to ECoV was detected in 17 of 29 farms (58.6%). In most positive farms (11 of 17), exposure was identified in a single horse, while in six farms between two and 12 horses tested positive, with the positive farm prevalence ranging between 12.5% and 37.5% (Figure 1).

### 3.2. Risk Factors Associated with Exposure to ECoV

ECoV seroprevalence was higher in horses residing in central Israel than in horses from the north or south (odds ratio (OR) = 6.08, *p* < 0.001, Table 1), and was lower in horses kept in pastures (OR = 0.19, *p* = 0.015, Table 1). Although the median age was slightly higher in seropositive horses (13 years versus 11 years), the distribution of ages did not differ statistically between seropositive and seronegative horses (*p* = 0.055). A significant interaction was found between the geographical area and housing management, as the vast majority (63 of 64) of pastured horses resided in the North. The only factor found to be significantly associated with ECoV exposure in the multivariable model was horses residing in central Israel (*p* < 0.001).

## 4. Discussion

The seroprevalence for ECoV in horses in Israel was 12.3% (CI 8.98–16.33%) and varied between geographical locations, similarly to the report from the USA, where ECoV seroprevalence was 9.6%, and varied between 4.0% and 19.7% in different states [9].

In this study, the rate of exposure was significantly higher in horses residing in central Israel and significantly lower in horses housed on pastures. Considering that the majority of the horses housed on pastures were from the North of Israel, these two factors were associated, and it is not surprising that the seroprevalence was lower in the North. It is possible that the orofecal route of transmission is limited in horses on pastures, due to a lower density, open air, and natural ventilation. As previously described, increased density (i.e., small paddocks compared to pasture) is likely to increase orofecal transmission of ECoV [8,9,13,15].

While the majority of horses in central Israel live in stalls or paddocks, it is relevant to mention that some large farms experience a relatively high turnover of horses due to leisure and competition considerations. It is possible that the relatively higher density in this housing practice, together with the increased introduction of new horses (occasionally imported from the USA or EU), increase the risk of exposure to carrier individuals or environmental contamination [9,21].

The horse population in this study was comprised of apparently healthy horses with no reported recent history of illness. However, the clinical significance of ECoV infection in this population is unknown. In addition, the use of a serologic assay in a single blood test does not allow to estimate the time of exposure and cannot rule out past or future clinical signs. Moreover, since it is unknown how long after exposure the horses remain seropositive (assuming no repeated exposure), it is impossible to date the timing of exposure and associate it with the medical history of these horses. The fact that the majority of horses infected with ECoV remain subclinical or experienced only mild non-specific clinical signs, such as fever or enteric disease [22], also complicates any retrospective assessment of possible infection in “positive” farms.

Similar to other β-coronaviruses, ECoV is considered to originate from bats [22], possibly descending from BCoV or a rat coronavirus [22]. Thus, while the introduction of ECoV to Israel may have resulted from international horse trade and horses travelling between the USA, the EU and Israel, a possible local exposure to a bat coronavirus should also be considered. Regardless of their housing management, most horses in Israel are not isolated from the environment, either rural or urban (walls and windows are usually freely open to the environment); therefore, such exposure is highly plausible. Future characterization of the Israeli ECoV strains is needed to further investigate its possible origin and its enzootic potential or zoonotic risk.

The amino acid sequence of the ECoV Spike protein is considered to be highly conserved [7]. ECoV is closely related to the BCoV and camel coronavirus (HKU23) [23,24], and horses vaccinated with the BCoV vaccine were demonstrating some extent of neutralizing immunity against ECoV [24]. In the same year that the study serum samples were collected (2018), 20/52 (38.5%) of the fecal and intestinal samples from cattle that were tested for BCoV in the Kimron Veterinary Institute were positive by qPCR [25]. Since some horse farms in Israel are located near bovine stocks (dairy and beef pastures), exposure of horses to BCoV is possible and needs to be further investigated. On one of the farms in the south of Israel, where 3/24 horses tested seropositive for ECoV, horses were housed in paddocks within a large alpaca farm. A recent study indicated limited circulation of MERS-CoV in camelids in Israel [18]. Little is known of the possible circulation of coronaviruses between mammalian hosts and molecular studies are warranted to investigate possible common sources of infection in different animal species.

The ECoV S protein-based ELISA used in this study has a sensitivity of 100% and a specificity of 90.48% when using an OD cutoff of 1.958 [16]. Nucleocapsid (N) protein-based serological assays are more often associated with cross-reactivity than S protein-based assays [26], and yet, without molecular confirmation of ECoV in horses in Israel, cross-reactivity cannot be completely dismissed and the results of this study should be interpreted with caution.

This study indicates that horses in Israel were exposed to coronavirus, most probably to ECoV. Therefore, ECoV should be included in the differential diagnosis list of pathogens in cases of adult horses with anorexia, lethargy, fever and gastrointestinal signs in Israel. Continuous surveillance, the isolation and characterization of isolates, and the identification of the origin of infection is needed to further characterize the clinical and epidemiological significance of ECoV in horse populations.

## 5. Conclusions

Exposure of horses in Israel to coronavirus, most probably to ECoV, is low, with higher seroprevalence in horses residing in central Israel. So far, exposure of horses to ECoV in the Middle East was only reported from Saudi Arabia. Further surveillance and molecular characterization of ECoV in horses from Israel is needed to confirm its presence in the area.

## Figures and Tables

**Table 1:** Univariable analysis of the risk factors associated with exposure to Equine coronavirus (ECoV) for horses in Israel, 2018.

Variable Category		*N*	ECoV-Positive (%)	OR (95% CI)	*p*
Area	North	150	9 (6%)	ref	-
-	Center	93	26 (28%)	6.08 (2.57–15.48)	<0.001
-	South	90	6 (6.7%)	1.12 (0.32–3.66)	1
Breed	Mixed	156	18 (11.5%)	ref	-
-	Pure bred	177	23 (13%)	1.15 (0.56–2.35)	0.74
Sex	Mare	161	21 (13%)	ref	-
-	Stallion	8	2 (25%)	2.22 (0.21–13.45)	0.298
-	Gelding	164	18 (11%)	0.82 (0.39–1.7)	0.611
Housing	Stall	136	20 (14.7%)	ref	-
-	Paddock	133	19 (14.3%)	0.97 (0.46–2.02)	1
-	Pasture	64	2 (3.1%)	0.19 (0.02–0.82)	0.015

## Data Availability

Data is contained within the article or Supplementary Material.

## Supplementary Materials

The following are available online at https://www.mdpi.com/2076-2615/11/3/894/s1, Table S1: Study population of horses from 29 farms throughout Israel.
