# Supplementary material for: Seroprevalence and Risk Factors for Exposure to Equine Coronavirus in Apparently Healthy Horses in Israel
Source: Animals (Basel). 2021 Mar 21;11(3):894. doi: 10.3390/ani11030894 (PMC8004030; doi:10.3390/ani11030894)
Supplement: Supplementary file 1 [file animals-11-00894-s001.pdf]

Farm	Farm Location	Area	Horse No	Age	Breed	Sex	Housing	ECoV
1	Ramat Gan	Center	E2	15.0	Pony	Mare	Stall	1
1	Ramat Gan	Center	E3	12.0	Mixed	Mare	Stall	1
1	Ramat Gan	Center	E5	13.0	Mixed	Mare	Stall	0
1	Ramat Gan	Center	E8	12.0	Mixed	Mare	Stall	0
1	Ramat Gan	Center	E16	13.0	Mixed	Mare	Stall	0
1	Ramat Gan	Center	E18	20.0	Mixed	Gelding	Stall	0
1	Ramat Gan	Center	E27	17.0	Mixed	Gelding	Stall	1
1	Ramat Gan	Center	E29	8.0	Draft	Gelding	Stall	0
1	Ramat Gan	Center	E32	20.0	Mixed	Mare	Stall	1
1	Ramat Gan	Center	E33	6.0	Draft	Gelding	Stall	0
1	Ramat Gan	Center	E34	11.0	Mixed	Gelding	Stall	1
1	Ramat Gan	Center	E35	17.0	Mixed	Gelding	Stall	0
1	Ramat Gan	Center	E36	12.0	Mixed	Gelding	Stall	1
1	Ramat Gan	Center	E37	22.0	Pony	Stallion	Stall	1
1	Ramat Gan	Center	E38	4.0	QH	Gelding	Stall	0
1	Ramat Gan	Center	E39	3.5	Draft	Gelding	Stall	0
1	Ramat Gan	Center	E40	12.0	Mixed	Mare	Stall	0
1	Ramat Gan	Center	E41	9.0	Ar	Gelding	Stall	0
1	Ramat Gan	Center	E42	9.0	Pony	Mare	Stall	0
1	Ramat Gan	Center	E43	14.0	Mixed	Mare	Stall	1
1	Ramat Gan	Center	E44	15.0	Mixed	Gelding	Stall	0
1	Ramat Gan	Center	E45	13.0	QH	Gelding	Stall	0
1	Ramat Gan	Center	E46	15.0	QH	Gelding	Stall	1
1	Ramat Gan	Center	E47	8.0	Pony	Mare	Stall	1
1	Ramat Gan	Center	E48	24.0	Pony	Gelding	Stall	0
1	Ramat Gan	Center	E50	14.0	TWH	Mare	Stall	0
1	Ramat Gan	Center	E51	18.0	WB	Mare	Stall	1
1	Ramat Gan	Center	E52	10.0	WB	Gelding	Stall	0
1	Ramat Gan	Center	E53	15.0	Mixed	Gelding	Stall	0
1	Ramat Gan	Center	E54	8.0	Pony	Gelding	Stall	1
1	Ramat Gan	Center	E55	11.0	QH	Gelding	Stall	0
1	Ramat Gan	Center	E56	15.0	Mixed	Gelding	Stall	0
2	Givat Haim I	Center	GH1	19.0	Ar	Mare	Paddock	1
2	Givat Haim I	Center	GH2	19.0	Ar	Mare	Paddock	0
2	Givat Haim I	Center	GH3	7.0	Ar	Mare	Paddock	0
2	Givat Haim I	Center	GH4	14.0	Ar	Stallion	Paddock	1
2	Givat Haim I	Center	GH5	13.0	Ar	Mare	Paddock	0
2	Givat Haim I	Center	GH6	16.0	Ar	Mare	Paddock	0
2	Givat Haim I	Center	GH7	13.0	Ar	Mare	Paddock	1
2	Givat Haim I	Center	GH8	10.0	Ar	Mare	Paddock	0
2	Givat Haim I	Center	GH9	15.0	Ar	Mare	Paddock	0
2	Givat Haim I	Center	GH10	12.0	Ar	Mare	Paddock	0
2	Givat Haim I	Center	GH11	5.0	Ar	Mare	Paddock	0
2	Givat Haim I	Center	GH12	13.0	Ar	Mare	Paddock	0
2	Givat Haim I	Center	GH13	17.0	WB	Gelding	Paddock	0
3	Ein Vered	Center	EV1	3.0	Mixed	Gelding	Paddock	0

3	Ein Vered	Center	EV2	12.0	Mixed	Gelding	Paddock	0
3	Ein Vered	Center	EV3	6.0	Mixed	Gelding	Paddock	0
3	Ein Vered	Center	EV4	8.0	Mixed	Gelding	Paddock	1
3	Ein Vered	Center	EV5	17.0	Mixed	Gelding	Paddock	0
3	Ein Vered	Center	EV6	10.0	Mixed	Gelding	Paddock	0
3	Ein Vered	Center	EV7	17.0	TB	Gelding	Paddock	0
4	Raanana	Center	FR1	14.0	Mixed	Mare	Paddock	0
4	Raanana	Center	FR2	10.0	Mixed	Gelding	Paddock	0
4	Raanana	Center	FR3	6.0	Mixed	Mare	Paddock	1
4	Raanana	Center	FR4	38.0	Mixed	Mare	Paddock	0
4	Raanana	Center	FR5	5.5	Mixed	Mare	Paddock	0
4	Raanana	Center	FR6	5.0	Pony	Gelding	Paddock	0
4	Raanana	Center	FR7	22.0	Pony	Gelding	Paddock	0
4	Raanana	Center	FR8	20.0	Pony	Mare	Paddock	1
4	Raanana	Center	FR9	15.0	Mixed	Gelding	Paddock	0
4	Raanana	Center	FR10	26.0	Mixed	Gelding	Paddock	0
4	Raanana	Center	FR11	26.0	Mixed	Gelding	Paddock	0
4	Raanana	Center	FR12	12.0	Pony	Mare	Paddock	0
4	Raanana	Center	FR13	22.0	Mixed	Gelding	Paddock	1
4	Raanana	Center	FR14	47.0	Mixed	Gelding	Paddock	0
4	Raanana	Center	FR15	15.0	Mixed	Gelding	Paddock	0
4	Raanana	Center	FR16	17.0	Mixed	Gelding	Paddock	0
4	Raanana	Center	FR17	11.0	Mixed	Gelding	Paddock	1
4	Raanana	Center	FR18	17.0	Mixed	Gelding	Paddock	1
4	Raanana	Center	FR19	14.0	Mixed	Gelding	Paddock	0
4	Raanana	Center	FR20	25.0	TB	Gelding	Paddock	1
5	Ora	Center	G4	12.0	Mixed	Gelding	Stall	0
5	Ora	Center	G5	9.0	Mixed	Gelding	Stall	0
5	Ora	Center	G6	16.0	QH	Gelding	Stall	0
5	Ora	Center	G15	8.0	QH	Mare	Stall	0
5	Ora	Center	G16	8.0	QH	Gelding	Stall	0
6	Beer Tuvia	South	AL1	9.0	QH	Gelding	Stall	0
6	Beer Tuvia	South	AL2	8.0	QH	Gelding	Stall	0
6	Beer Tuvia	South	AL3	8.0	QH	Gelding	Stall	0
6	Beer Tuvia	South	AL4	5.0	QH	Gelding	Stall	0
6	Beer Tuvia	South	AL5	8.0	QH	Gelding	Stall	0
6	Beer Tuvia	South	AL6	13.0	QH	Gelding	Stall	0
6	Beer Tuvia	South	AL7	4.0	QH	Gelding	Stall	0
6	Beer Tuvia	South	AL8	14.0	QH	Gelding	Stall	0
6	Beer Tuvia	South	AL9	9.0	QH	Gelding	Stall	0
6	Beer Tuvia	South	AL10	15.0	QH	Mare	Stall	0
6	Beer Tuvia	South	AL11	13.0	QH	Mare	Stall	0
6	Beer Tuvia	South	AL12	14.0	QH	Gelding	Stall	0
6	Beer Tuvia	South	AL13	19.0	QH	Mare	Stall	0
6	Beer Tuvia	South	AL14	7.0	QH	Gelding	Stall	0
6	Beer Tuvia	South	AL15	17.0	TB	Mare	Stall	0
7	Kidron	Center	KN1	11.0	Ar	Mare	Paddock	0

7	Kidron	Center	KN2	7.0	Ar	Mare	Paddock	0
7	Kidron	Center	KN3	19.0	Ar	Mare	Paddock	0
7	Kidron	Center	KN4	18.0	Ar	Mare	Paddock	0
7	Kidron	Center	KN5	20.0	Ar	Mare	Paddock	0
7	Kidron	Center	KN6	15.0	Ar	Mare	Paddock	1
7	Kidron	Center	KN7	10.0	Ar	Mare	Paddock	0
7	Kidron	Center	KN8	8.0	Ar	Mare	Paddock	0
7	Kidron	Center	KN9	9.0	Ar	Mare	Paddock	0
7	Kidron	Center	KN10	8.0	Ar	Mare	Paddock	0
7	Kidron	Center	KN11	15.0	Ar	Mare	Paddock	0
7	Kidron	Center	KN12	12.0	Ar	Mare	Paddock	0
7	Kidron	Center	KN13	7.0	Ar	Mare	Paddock	1
7	Kidron	Center	KN14	9.0	Ar	Mare	Paddock	1
7	Kidron	Center	KN15	9.0	Ar	Mare	Paddock	0
7	Kidron	Center	KN16	3.0	Ar	Mare	Paddock	1
8	Brehya	South	BR1	9.0	Mixed	Gelding	Stall	1
8	Brehya	South	BR2	5.0	Mixed	Gelding	Stall	0
8	Brehya	South	BR3	15.0	Mixed	Mare	Stall	0
8	Brehya	South	BR4	10.0	Mixed	Gelding	Stall	0
8	Brehya	South	BR5	18.5	Mixed	Gelding	Stall	0
8	Brehya	South	BR6	10.0	Mixed	Gelding	Stall	0
8	Brehya	South	BR7	7.0	Mixed	Gelding	Stall	0
8	Brehya	South	BR8	14.0	Mixed	Mare	Stall	0
8	Brehya	South	BR9	7.0	Mixed	Gelding	Stall	1
8	Brehya	South	BR10	20.0	Mixed	Gelding	Stall	0
9	Gilat	South	HG1	2.0	QH	Mare	Stall	0
9	Gilat	South	HG2	4.0	Mixed	Mare	Stall	0
9	Gilat	South	HG3	11.0	Mixed	Gelding	Stall	0
9	Gilat	South	HG4	15.0	QH	Gelding	Stall	0
9	Gilat	South	HG5	20.0	App	Mare	Stall	0
9	Gilat	South	HG6	15.0	QH	Gelding	Stall	0
9	Gilat	South	HG7	9.0	QH	Mare	Stall	1
9	Gilat	South	HG8	17.0	QH	Mare	Stall	0
9	Gilat	South	HG9	4.0	QH	Gelding	Stall	0
9	Gilat	South	HG10	7.0	QH	Mare	Paddock	0
9	Gilat	South	HG11	10.0	Mixed	Gelding	Paddock	0
9	Gilat	South	HG12	13.0	QH	Mare	Paddock	0
9	Gilat	South	HG13	16.0	QH	Mare	Paddock	0
9	Gilat	South	HG14	12.0	Mixed	Mare	Stall	0
10	Sde Araham	South	SA1	12.0	Andalus	Gelding	Stall	0
10	Sde Araham	South	SA2	11.0	Mixed	Gelding	Stall	0
10	Sde Araham	South	SA3	16.0	WB	Gelding	Stall	0
10	Sde Araham	South	SA4	16.0	Pony	Mare	Stall	0
10	Sde Araham	South	SA5	14.0	Pony	Mare	Stall	0
10	Sde Araham	South	SA6	8.0	Pony	Gelding	Stall	0
10	Sde Araham	South	SA7	10.0	Pony	Gelding	Stall	0
10	Sde Araham	South	SA8	10.0	WB	Gelding	Stall	0

10	Sde Araham	South	SA9	16.0	WB	Gelding	Stall	0
10	Sde Araham	South	SA10	19.0	WB	Gelding	Stall	0
11	Mishmar Hanegev	South	MH1	5.0	Mixed	Mare	Stall	0
11	Mishmar Hanegev	South	MH2	0.5	Mixed	Mare	Stall	0
11	Mishmar Hanegev	South	MH3	11.0	Mixed	Mare	Stall	0
11	Mishmar Hanegev	South	MH4	18.0	Mixed	Mare	Stall	0
11	Mishmar Hanegev	South	MH5	5.0	Mixed	Stallion	Paddock	0
12	Mitzpe Ramon	South	MR1	12.0	Mixed	Gelding	Paddock	0
12	Mitzpe Ramon	South	MR2	6.0	Ar	Mare	Paddock	0
12	Mitzpe Ramon	South	MR3	17.0	Ar	Gelding	Paddock	1
12	Mitzpe Ramon	South	MR4	5.0	Pony	Gelding	Paddock	0
12	Mitzpe Ramon	South	MR5	7.0	Ar	Gelding	Paddock	1
12	Mitzpe Ramon	South	MR6	6.0	Ar	Mare	Paddock	0
12	Mitzpe Ramon	South	MR7	11.0	Mixed	Gelding	Paddock	0
12	Mitzpe Ramon	South	MR8	5.0	Mixed	Mare	Paddock	0
12	Mitzpe Ramon	South	MR9	7.0	Mixed	Gelding	Paddock	0
12	Mitzpe Ramon	South	MR10	16.0	Mixed	Gelding	Paddock	0
12	Mitzpe Ramon	South	MR11	16.0	Mixed	Mare	Paddock	1
12	Mitzpe Ramon	South	MR12	38.0	Mixed	Gelding	Paddock	0
12	Mitzpe Ramon	South	MR14	7.0	Mixed	Mare	Paddock	0
12	Mitzpe Ramon	South	MR15	23.0	Mixed	Mare	Paddock	0
12	Mitzpe Ramon	South	MR16	11.0	Ar	Gelding	Paddock	0
12	Mitzpe Ramon	South	MR17	18.0	Mixed	Gelding	Paddock	0
12	Mitzpe Ramon	South	MR18	15.0	Mixed	Gelding	Paddock	0
12	Mitzpe Ramon	South	MR19	17.0	Ar	Gelding	Paddock	0
12	Mitzpe Ramon	South	MR20	13.0	Mixed	Gelding	Paddock	0
12	Mitzpe Ramon	South	MR21	12.0	Mixed	Mare	Paddock	0
12	Mitzpe Ramon	South	MR22	6.0	Mixed	Gelding	Paddock	0
12	Mitzpe Ramon	South	MR23	15.0	Mixed	Gelding	Paddock	0
12	Mitzpe Ramon	South	MR24	1.5	Ar	Stallion	Paddock	0
12	Mitzpe Ramon	South	MR25	12.0	Ar	Stallion	Paddock	0
13	Ein Yahav	South	Q1	7.0	Mixed	Mare	Paddock	0
13	Ein Yahav	South	Q2	12.0	Mixed	Mare	Paddock	0
13	Ein Yahav	South	Q4	10.0	Mixed	Mare	Paddock	0
13	Ein Yahav	South	Q9	20.0	Mixed	Gelding	Paddock	0
14	Kalia	South	H2	17.0	QH	Mare	Paddock	0
14	Kalia	South	H4	13.0	QH	Mare	Paddock	0
14	Kalia	South	H5	12.0	QH	Mare	Paddock	0
14	Kalia	South	H6	11.0	Mixed	Gelding	Paddock	0
14	Kalia	South	H7	17.0	PH	Gelding	Paddock	0
14	Kalia	South	H12	15.0	TWH	Mare	Paddock	0
14	Kalia	South	H13	8.0	QH	Gelding	Paddock	0
14	Kalia	South	H14	23.0	Mixed	Gelding	Pasture	0
15	Vered Hagalil	North	R1	12.0	Pony	Mare	Pasture	0
15	Vered Hagalil	North	R3	10.0	Mixed	Mare	Pasture	0
15	Vered Hagalil	North	R4	8.0	Mixed	Gelding	Pasture	0
15	Vered Hagalil	North	R7	8.0	Mixed	Gelding	Pasture	0

15	Vered Hagalil	North	R8	10.0	Mixed	Gelding	Pasture	0
15	Vered Hagalil	North	R9	9.0	QH	Gelding	Pasture	0
15	Vered Hagalil	North	R10	10.0	QH	Gelding	Pasture	0
15	Vered Hagalil	North	R11	8.0	Mixed	Gelding	Pasture	0
15	Vered Hagalil	North	R12	6.0	Mixed	Mare	Pasture	0
15	Vered Hagalil	North	R13	6.0	Mixed	Gelding	Pasture	0
15	Vered Hagalil	North	R14	10.0	QH	Mare	Pasture	0
15	Vered Hagalil	North	R15	12.0	QH	Mare	Pasture	0
15	Vered Hagalil	North	R16	10.0	QH	Gelding	Pasture	1
15	Vered Hagalil	North	R17	6.0	Mixed	Gelding	Pasture	0
15	Vered Hagalil	North	R18	20.0	Mixed	Gelding	Pasture	0
15	Vered Hagalil	North	R20	6.0	Mixed	Gelding	Pasture	0
15	Vered Hagalil	North	R21	4.0	Mixed	Gelding	Pasture	0
15	Vered Hagalil	North	R22	5.0	Mixed	Gelding	Pasture	0
15	Vered Hagalil	North	R23	5.0	Mixed	Gelding	Pasture	0
15	Vered Hagalil	North	R24	8.0	Mixed	Gelding	Pasture	0
15	Vered Hagalil	North	R25	9.0	Mixed	Mare	Pasture	0
15	Vered Hagalil	North	R26	8.0	Mixed	Gelding	Paddock	0
16	Binyamina	North	B1	19.0	TB	Gelding	Paddock	0
16	Binyamina	North	B2	18.0	Mixed	Mare	Paddock	0
16	Binyamina	North	B3	20.0	Mixed	Mare	Paddock	0
16	Binyamina	North	B4	6.0	MFT	Mare	Paddock	0
16	Binyamina	North	B9	4.0	QH	Gelding	Paddock	1
16	Binyamina	North	B10	20.0	Mixed	Mare	Paddock	0
16	Binyamina	North	B11	12.0	PH	Mare	Paddock	0
16	Binyamina	North	B12	1.2	QH	Mare	Paddock	0
16	Binyamina	North	B13	4.0	PH	Mare	Paddock	0
16	Binyamina	North	B14	18.0	QH	Mare	Paddock	0
16	Binyamina	North	B15	14.0	PH	Mare	Paddock	0
16	Binyamina	North	B16	8.0	TWH	Gelding	Paddock	0
16	Binyamina	North	B17	5.0	MFT	Gelding	Paddock	0
16	Binyamina	North	B18	20.0	WB	Gelding	Paddock	0
16	Binyamina	North	B19	18.0	PH	Gelding	Paddock	0
17	Ein Harod	North	N1	16.0	Mixed	Mare	Paddock	0
17	Ein Harod	North	N2	14.0	Mixed	Mare	Paddock	0
17	Ein Harod	North	N5	20.0	Mixed	Gelding	Paddock	0
17	Ein Harod	North	N6	5.0	Mixed	Mare	Paddock	0
17	Ein Harod	North	N7	11.0	Mixed	Mare	Paddock	0
17	Ein Harod	North	N12	10.0	QH	Mare	Paddock	0
17	Ein Harod	North	N13	5.0	WB	Stallion	Paddock	0
17	Ein Harod	North	N14	15.0	WB	Gelding	Paddock	0
17	Ein Harod	North	N15	2.5	WB	Mare	Stall	0
18	Kfar Szold	North	KS1	5.0	QH	Gelding	Stall	0
18	Kfar Szold	North	KS2	9.0	Mixed	Mare	Stall	0
18	Kfar Szold	North	KS3	10.0	QH	Gelding	Stall	0
18	Kfar Szold	North	KS4	6.0	Mixed	Mare	Stall	0
18	Kfar Szold	North	KS5	13.0	PH	Gelding	Stall	1

18	Kfar Szold	North	KS6	8.0	TWH	Gelding	Stall	0
18	Kfar Szold	North	KS7	4.5	Mixed	Gelding	Stall	0
19	Ein dor Galit	North	ED1	23.0	WB	Gelding	Stall	0
19	Ein dor Galit	North	ED2	15.0	Mixed	Gelding	Stall	1
19	Ein dor Galit	North	ED3	24.0	Mixed	Mare	Stall	0
19	Ein dor Galit	North	ED4	11.0	Pony	Mare	Stall	0
19	Ein dor Galit	North	ED5	1.5	Mixed	Mare	Stall	0
19	Ein dor Galit	North	ED6	10.0	QH	Mare	Pasture	0
20	Nov	North	L3	9.0	Mixed	Gelding	Pasture	0
20	Nov	North	L4	9.0	Mixed	Gelding	Pasture	0
20	Nov	North	L5	12.0	Mixed	Mare	Pasture	0
20	Nov	North	L6	5.0	Mixed	Mare	Pasture	0
20	Nov	North	L7	9.0	Mixed	Gelding	Pasture	0
20	Nov	North	L10	6.0	Mixed	Mare	Pasture	0
20	Nov	North	L11	16.0	App	Mare	Stall	0
21	Abirim	North	AB1	14.0	Mixed	Mare	Stall	1
21	Abirim	North	AB2	7.0	TWH	Mare	Stall	0
21	Abirim	North	AB3	10.0	Mixed	Gelding	Stall	0
21	Abirim	North	AB5	10.0	App	Gelding	Pasture	0
22	Misgav Am	North	MV1	1.0	TWH	Gelding	Pasture	0
22	Misgav Am	North	MV2	1.5	TWH	Gelding	Pasture	0
22	Misgav Am	North	MV3	12.5	Mixed	Mare	Stall	0
22	Misgav Am	North	MV4	2.5	Mixed	Mare	Stall	0
22	Misgav Am	North	MV5	5.5	Mixed	Mare	Pasture	0
22	Misgav Am	North	MV6	3.7	Mixed	Mare	Pasture	0
22	Misgav Am	North	MV7	2.5	Mixed	Gelding	Stall	0
23	Beit Zera	North	EK1	14.0	TWH	Mare	Stall	0
23	Beit Zera	North	EK2	9.0	TWH	Mare	Stall	0
23	Beit Zera	North	EK3	5.0	TWH	Mare	Stall	0
23	Beit Zera	North	EK4	18.0	TWH	Mare	Stall	0
23	Beit Zera	North	EK5	2.5	TWH	Stallion	Stall	0
24	Kfar Vradim Ma'a lc	North	KV1	14.0	Mixed	Mare	Stall	1
24	Kfar Vradim Ma'a lc	North	KV2	17.0	Mixed	Gelding	Stall	0
24	Kfar Vradim Ma'a lc	North	KV3	15.0	Mixed	Mare	Stall	0
24	Kfar Vradim Ma'a lc	North	KV4	18.0	QH	Gelding	Stall	0
24	Kfar Vradim Ma'a lc	North	KV5	15.0	QH	Mare	Stall	0
24	Kfar Vradim Ma'a lc	North	KV6	11.0	Mixed	Gelding	Stall	0
24	Kfar Vradim Ma'a lc	North	KV7	13.0	Mixed	Gelding	Stall	0
24	Kfar Vradim Ma'a lc	North	KV8	14.0	Mixed	Mare	Stall	0
24	Kfar Vradim Ma'a lc	North	KV9	20.0	Mixed	Gelding	Stall	0
24	Kfar Vradim Ma'a lc	North	KV10	10.0	Mixed	Mare	Paddock	0
25	Alonim	North	Z1	8.0	QH	Mare	Paddock	0
25	Alonim	North	Z2	15.0	QH	Gelding	Paddock	0
25	Alonim	North	Z3	19.0	Mixed	Mare	Paddock	0
25	Alonim	North	Z4	10.0	QH	Mare	Paddock	0
25	Alonim	North	Z5	13.0	QH	Mare	Paddock	0
25	Alonim	North	Z6	10.0	QH	Mare	Paddock	0

25	Alonim	North	Z7	17.0	QH	Mare	Paddock	0
25	Alonim	North	Z8	10.0	QH	Mare	Paddock	0
25	Alonim	North	Z9	17.0	QH	Mare	Paddock	1
25	Alonim	North	Z10	3.0	Mixed	Gelding	Paddock	0
25	Alonim	North	Z11	12.0	Mixed	Gelding	Paddock	0
25	Alonim	North	Z12	4.0	QH	Gelding	Paddock	0
25	Alonim	North	Z13	11.0	QH	Gelding	Stall	0
26	Megadim	North	Y1	15.0	QH	Gelding	Stall	0
26	Megadim	North	Y2	9.0	Mixed	Gelding	Stall	0
26	Megadim	North	Y3	8.0	Mixed	Gelding	Stall	0
26	Megadim	North	Y4	11.0	Mixed	Gelding	Stall	0
26	Megadim	North	Y5	15.0	QH	Mare	Stall	0
26	Megadim	North	Y6	16.0	PH	Mare	Stall	0
26	Megadim	North	Y7	7.0	QH	Mare	Stall	0
26	Megadim	North	Y8	11.0	Mixed	Gelding	Pasture	0
27	Kerem Maharal	North	A8	3.0	Mixed	Gelding	Pasture	0
27	Kerem Maharal	North	A9	5.0	Mixed	Mare	Pasture	0
27	Kerem Maharal	North	A11	4.0	Mixed	Gelding	Pasture	0
27	Kerem Maharal	North	A12	2.0	Mixed	Gelding	Pasture	0
27	Kerem Maharal	North	A19		Mixed	Gelding	Pasture	0
27	Kerem Maharal	North	A20		Mixed	Mare	Pasture	0
27	Kerem Maharal	North	A21		Mixed	Gelding	Pasture	0
27	Kerem Maharal	North	A22		Mixed	Mare	Pasture	0
28	Ein Hamifraz	North	EH1	2.0	WB	Mare	Stall	0
28	Ein Hamifraz	North	EH2	9.0	Mixed	Gelding	Stall	0
28	Ein Hamifraz	North	EH3	1.0	Mixed	Stallion	Stall	0
28	Ein Hamifraz	North	EH4	4.0	Mixed	Gelding	Stall	0
28	Ein Hamifraz	North	EH5	3.0	WB	Mare	Stall	0
28	Ein Hamifraz	North	EH6	13.0	WB	Mare	Stall	1
28	Ein Hamifraz	North	EH7	0.5	WB	Mare	Stall	0
28	Ein Hamifraz	North	EH8	4.0	Mixed	Mare	Stall	0
29	Cabri	North	CB1	15.0	Ar	Mare	Pasture	0
29	Cabri	North	CB2	13.0	Ar	Mare	Pasture	0
29	Cabri	North	CB3	9.0	Mixed	Mare	Pasture	1
29	Cabri	North	CB4	11.0	Mixed	Mare	Pasture	0
29	Cabri	North	CB5	11.0	Ar	Mare	Pasture	0
29	Cabri	North	CB6	11.0	Ar	Mare	Pasture	0
29	Cabri	North	CB7	11.0	Ar	Gelding	Pasture	0
29	Cabri	North	CB8	15.0	Mixed	Mare	Pasture	0
29	Cabri	North	CB9	9.0	Pony	Gelding	Pasture	0
29	Cabri	North	CB10	12.0	Mixed	Mare	Pasture	0
29	Cabri	North	CB11	16.0	Mixed	Mare	Pasture	0
29	Cabri	North	CB12	12.0	QH	Mare	Pasture	0
29	Cabri	North	CB13	13.0	QH	Mare	Pasture	0
29	Cabri	North	CB14	11.0	Mixed	Gelding	Pasture	0
29	Cabri	North	CB15	15.0	Pony	Gelding	Pasture	0
29	Cabri	North	CB16	21.0	Ar	Mare	Pasture	0

29	Cabri	North	CB17	21.0	Ar	Mare	Pasture	0
29	Cabri	North	CB18	5.0	QH	Mare	Pasture	0
29	Cabri	North	CB19	14.0	QH	Gelding	Pasture	0
29	Cabri	North	CB20	13.0	Ar	Mare	Pasture	0
29	Cabri	North	CB21	11.0	Mixed	Gelding	Pasture	0
